# Supplementary material for: Archaean multi-stage magmatic underplating drove formation of continental nuclei in the North China Craton
Source: Nat Commun. 2024 Jul 24;15:6231. doi: 10.1038/s41467-024-50435-5 (PMC11266541; doi:10.1038/s41467-024-50435-5)
Supplement: Supplementary file 1 — Supplementary Information [file 41467_2024_50435_MOESM1_ESM.pdf]

Archaean multi-stage magmatic underplating drove formation of continental nuclei in the North China Craton

Jin Liu[1,2], Richard M. Palin[2*], Ross N. Mitchell[3,4], Zhenghong Liu[1,5], Jian Zhang[6], Zhongshui Li[7], Changquan Cheng[8], Hongxiang Zhang[1]

[1]College of Earth Sciences, Jilin University, 2199 Jianshe Street, Changchun, 130061, China

[2]Department of Earth Sciences, University of Oxford, South Parks Road, Oxford, OX1 3AN United Kingdom

[3]State Key Laboratory of Lithospheric Evolution, Institute of Geology and Geophysics, Chinese Academy of Sciences, Beijing, 100029, China.

[4]College of Earth and Planetary Sciences, University of Chinese Academy of Sciences, Beijing, 100049, China

[5]Key Laboratory of Mineral Resources Evaluation in Northeast Asia, Ministry of Natural Resources, Changchun 130061, China

[6]Department of Earth Sciences, The University of Hong Kong, Pokfulam Road, Hong Kong, China

[7]College of Exploration and Geomatics Engineering, Changchun Institute of Technology, Changchun, 130103, China

[8]School of Earth Sciences and Engineering, Sun Yat-sen University, Zhuhai 519000, China

*E-mail: richard.palin@earth.ox.ac.uk

Supplementary Methods

Zircon LA–ICP–MS U–Pb dating

Zircon grains were separated using conventional density and magnetic methods. The ones with minimal cracks and radiation damage were embedded in epoxy resin and polished to about half their thickness. Cathodoluminescence (CL) imaging was conducted on the resin-embedded samples to reveal the internal structure of the zircons, a process carried out at Nanjing Hongchuang Geological Exploration Technology Service Co., Ltd. Prior to analysis, the resin-embedded sample was cleaned using ultrasonic waves and subsequently coated with a layer of carbon. The CL images were captured using a Tescan MIRA3 LM instrument, which is equipped with a CL detector. A consistent acceleration voltage and current of 7kV were applied throughout the process. Each CL image was gathered over an 80-second accumulation period, with approximately 15% overlap with adjacent images to ensure a seamless panoramic stitch.

The zircon LA–ICP–MS U–Pb analysis of samples 21LJ38-1, 21LJ39-1, 21LJ35-1, 21LJ06-3, 21LJ18-1, and 21LJ19-1 was conducted using a NWR193 laser-ablation microprobe from Elemental Scientific Lasers LLC. This was attached to an Analytikjena PlasmaQuant MS quadrupole ICP–MS at Yanduzhongshi Geological Analysis Laboratories Ltd. To adjust sensitivity during the laser ablation process, helium was used as the carrier gas and argon as the compensation gas. These gases were mixed at a Y-shaped junction before entering the ICP. Each time-resolved analysis included an approximately 20 to 30-second blank signal and a 40-second sample signal. Data processing was performed offline using the ICPMSDataCal software[1]. This included selecting samples and blank signals, correcting instrument sensitivity drift, and calculating elemental contents as well as U–Th–Pb

isotope ratios and ages. The standard 91500 (1062.4 ± 0.4 Ma; Wiedenbeck et al. [2]) and NIST 610 were used for external calibration of the U–Pb ages and trace element content calculations respectively. Corrections for instrument drift, mass bias, and fractionation of the U–Pb ratio were made using a standard-sample bracketing method. For quality control purposes, the zircon standard Plešovice (337.13 ± 0.37 Ma, Sláma et al. [3]) was analyzed after every ten unknown samples. Based on the actual situation, a testing denuded diameter of 30 μm was selected. The measured weighted mean ages of standards 91500 (1060.4 ± 2.9 Ma) and Plešovice (339.6 ± 1.4 Ma) matched the recommended values.

The analysis of samples 22BS20-3, 22BS15-1, 22BS19-1, 22BS23-1, 22BS27-1, 22BS28-1, 22BS29-1, 22BS32-1, 22BS18-4, 23HX22-1, 23HX23-1, 23HX28-1, and 23HX29-1 was carried out at the Key Laboratory of Mineral Resources Evaluation in Northeast Asia at Jilin University in Changchun, China. The LA–ICP–MS instrument used was equipped with a quadrupole ICP–MS (Agilent 7500c) and a 193-nm ArF Excimer laser (COMPexPro102 from Coherent DE), complete with an automatic positioning system. During the analysis process, helium (at a flow rate of 1.15 L/min) was used as the carrier gas to efficiently transport aerosols to the ICP while minimizing deposition around the ablation site and within the transport tube. Argon (at a flow rate of 600 ml/min) was used as a make-up gas and mixed with the carrier gas via a T-shaped connector before entering the ICP. For most analyses, the analytical spot size was set at 32 μm, with a laser energy density of 10 J/cm^2 and a repetition frequency of 8 Hz. The laser sampling procedure comprised a 30-second blank, followed by a 30-second sampling ablation, and concluded with a 2-minute flushing of the sample chamber post-ablation. The abundances of U, Th, and Pb were calibrated using ^{29}Si as an internal standard, with NIST610 glass serving as an external standard. Zircon standard 91500 was employed as the primary standard for age calibration, while zircon standard Plešovice was utilized as a secondary standard to monitor any deviations in age measurement or calculation. Analysis of zircon standard 91500 yielded a weighted mean ^{206}Pb/^{238}U age of 1062.2 ± 2.3 Ma, aligning with the recommended ^{206}Pb/^{238}U ages of 1062.4 ± 0.4 Ma for this standard within analytical errors[2]. Analysis of Plešovice yielded a weighted mean ^{206}Pb/^{238}U age of 335.9 ± 1.7 Ma matched the recommended values (337.13 ± 0.37 Ma; Sláma et al.[3]). Detailed descriptions of both the instrument and analytical procedure can be found in Yuan et al. [4]. Raw data processing was performed using ICPMSDataCal software[1].

The Zircon U–Pb dating of Sample Z2010-3 was performed using LA–ICP–MS at Nanjing Hongchuang Exploration Technology Service Co., Ltd. The laser ablation system used was the Resolution SE model from Applied Spectra, USA, equipped with an ATL (ATLEX 300) excimer laser and a Two Volume S155 ablation cell. This system was coupled with an Agilent 7900 ICPMS from Agilent, USA. For detailed tuning parameters, please refer to Thompson et al. [5]. The LA–ICP–MS tuning was carried out using a 50-micron diameter line scan at a speed of 3 μm/s on NIST 612, with an energy density of ~3.5 J/cm^2 and a repetition rate of 10 Hz. The gas flow was adjusted to achieve the highest sensitivity and the lowest oxide ratio (ThO/Th < 0.2%). P/A calibration was performed on NIST 610 using a 100-micron diameter line scan, with other laser parameters remaining consistent with those used during tuning. Each spot analysis underwent pre-ablation using five laser shots (~0.3 μm in depth) to eliminate potential surface contamination. The analysis was carried out on a 30 μm diameter spot at a frequency of 5 Hz and a fluence of 2.5 J/cm^2. Data reduction was performed using the Iolite software package[6]. Zircon standards 91500 and Plešovice were utilized as primary and secondary reference materials, respectively. Triplets of these standards were bracketed between multiple groups of unknown samples, each group containing between ten to twelve samples. Typically, sample signals were acquired for a duration of between 35 to 40 seconds following a gas background measurement lasting for about twenty seconds. An exponential function was used to calibrate downhole fractionation[6]. The measured ages of zircon standards 91500 (weighted mean age = 1060.3 ± 5.0 Ma) and Plešovice (weighted mean age = 338.2 ± 3.9 Ma) are indistinguishable from accepted reference values suggested by Wiedenbeck et al. [2] and Sláma et al. [3].

Uncertainties in individual analyses were presented with a 1σ error, while the weighted mean ages were computed at the 95% confidence level. The computation of weighted average ages and the creation of the

concordia diagram were accomplished using Isoplot 4.15 (ref. [7]).

Zircon SHRIMP U–Pb dating

The samples 21LJ39-1, 22BS20-3, and 22BS15-1 were also subjected to analysis using the SHRIMP II at the Beijing SHRIMP Center of the Chinese Academy of Geological Sciences (GAGS) in Beijing, China. The detailed analytical procedures and conditions followed those described by Williams [8]. During the analysis, the intensity of the primary O^{2-} ion beam from SHRIMP was set to 3–5nA, with beam spot sizes of 20 μm. Each analytical spot underwent a raster process for 2–3 minutes before analysis. Five scans were conducted on each spot to determine its age, with the entire process taking approximately fifteen minutes. Standard zircon of TEMORA ($^{206}Pb/^{208}U$ = 416.7 ± 1.3 Ma)[9] was utilized to correct the U, Th, and Pb contents as well as the ages of zircon. The measured ages of zircon standards TEMORA (weighted mean age = 417.3 ± 2.6 Ma) are consistent with the recommended age. Corrections for common lead were made using measured abundances of ^{204}Pb. The raw data and age assessments were processed using SQUID and IsoplotR software packages[7, 10].

Zircon Lu–Hf isotope analysis

In situ Hf isotope ratio analysis experiments were conducted using a Neptune Plus MC–ICP–MS (Thermo Fisher Scientific, Germany) in conjunction with a Geolas HD excimer ArF laser ablation system (Coherent, Göttingen, Germany). This system is housed at the Wuhan Sample Solution Analytical Technology Co., Ltd, Wuhan, China. Helium was used as the carrier gas within the ablation cell and was combined with argon (makeup gas) after the ablation cell. Small quantities of nitrogen were added to the argon makeup gas flow to enhance the sensitivity of Hf isotopes[11]. Compared to the standard setup, the addition of nitrogen, along with the use of the newly designed X skimmer cone and Jet sample cone in Neptune Plus, improved the signal intensity of Hf, Yb, and Lu by factors of 5.3, 4.0, and 2.4 respectively. All data were collected on zircon in single spot ablation mode at a spot size of 44 μm. The energy density of laser ablation used in this study was approximately 10 J cm^{-2}. Each measurement consisted of a 20-second acquisition of the background signal followed by a 50-second acquisition of the ablation signal. The operating conditions for the laser ablation system and the MC–ICP–MS instrument, as well as the analytical method, are consistent with those described by[11].

The primary challenge in achieving accurate in situ zircon Hf isotope determination by LA–MC–ICP–MS is the substantial isobaric interference from ^{176}Yb, and to a lesser extent, ^{176}Lu on ^{176}Hf. It has been demonstrated that the mass fractionation of Yb (β_{Yb}) is not constant over time, and the β_{Yb} obtained from solution introduction is not suitable for in situ zircon measurements[12]. Misestimating the β_{Yb} value would inevitably affect the accurate correction of ^{176}Yb, and consequently, the determined $^{176}Hf/^{177}Hf$ ratio. In this study, we used the β_{Yb} value directly obtained from the zircon sample itself in real-time. The $^{179}Hf/^{177}Hf$ and $^{173}Yb/^{171}Yb$ ratios were used to calculate the mass bias of Hf (β_{Hf}) and Yb (β_{Yb}), which were normalized to $^{179}Hf/^{177}Hf$ = 0.7325 and $^{173}Yb/^{171}Yb$ = 1.132685 (ref. [13]) using an exponential correction for mass bias. The interference of ^{176}Yb on ^{176}Hf was corrected by measuring the interference-free ^{173}Yb isotope and using $^{176}Yb/^{173}Yb$ = 0.79639 (ref. [13]) to calculate $^{176}Yb/^{177}Hf$. Similarly, the relatively minor interference of ^{176}Lu on ^{176}Hf was corrected by measuring the intensity of the interference-free ^{175}Lu isotope and using the recommended $^{176}Lu/^{175}Lu$ = 0.02656 (ref. [14]) to calculate $^{176}Lu/^{177}Hf$. Given their similar physicochemical properties, we used the mass bias of Yb (β_{Yb}) to calculate the mass fractionation of Lu. Off-line selection and integration of analyte signals, as well as mass bias calibrations, were performed using ICPMSDataCal[1].

To ensure the reliability of our analysis data, we simultaneously analyzed three international zircon standards: Plešovice, 91500, and GJ-1, alongside the actual samples. Plešovice was utilized for external standard calibration to optimize the analysis and test results further. Meanwhile, 91500 and GJ-1 served as secondary standards to monitor the quality of data correction. Lu-Hf data for these zircon standards are listed in Supplementary Data 3. The test $^{176}Hf/^{177}Hf$ values of Plešovice (weighted mean value = 0.282478), 91500 (weighted mean value = 0.282297), and GJ-1 (weighted mean value = 0.282005) (Supplementary Data 3) were consistent with the

recommended values within the error range (Plešovice = 0.282478 ± 8, 91500 = 0.282300 ± 11, and GJ-1 = 0.282009 ± 10) suggested by Zhang and Hu [15].

The $\varepsilon_{Hf(t)}$ values are calculated using ^{176}Lu decay constant (λ^{176}Lu) of 1.865 × 10^{-11}(ref. [16]) and the chondrite parameters used are ^{176}Hf/^{177}Hf = 0.282772 and ^{176}Lu/^{177}Hf = 0.0332 (ref. [17]). For the $\varepsilon_{Hf(t)}$ value calculations, apparent ^{207}Pb/^{206}Pb ages adopted for the zircon grains from the 3.3–3.2 Ga monzogranites and *c.* 2.5 Ga potassic granites with xenocrystic zircons. However, for the other granites aged between 2.7–2.5 Ga that do not contain xenocrystic zircons, the interpreted crystallization age of each sample was used. The zircon Hf two-stage depleted mantle model ages ($T_{DM}2$) were calculated using ratios of ^{176}Hf/^{177}Hf = 0.283251 and ^{176}Lu/^{177}Hf = 0.0384 for present depleted mantle suggested by Griffin et al. [18], and ^{176}Lu/^{177}Hf value of 0.022.

Major and trace element analyses

Whole-rock major and trace element analyses were conducted at Wuhan Sample Solution Analytical Technology Co., Ltd, China. The Zsx Primus II wavelength dispersive X-ray fluorescence spectrometer (XRF) was used to analyze the major elements. The sample pretreatment of whole rock major element analysis was made by melting method. The flux is a mixture of lithium tetraborate, lithium metaborate and lithium fluoride (45:10:5), Ammonium nitrate and lithium bromide were used as oxidant and release agent respectively. The melting temperature was 1050 ℃ and the melting time was 15 minutes. The test condition was set at a voltage of 50 kV and a current of 60 mA. The standard curves were derived using the national standard materials GBW07103, GBW07105, GBW07111, and GBW071112. The relative standard deviation (RSD) was less than 2%. An Agilent 7700e ICP-MS equipment was used to analyze the trace elements. A 50 mg sample powder (200 mesh) was dissolved in a 2ml HNO_3+HF (1:1) solution within a Teflon bomb. The Teflon bomb was subsequently heated up to 190 ℃ for more than 24 hours. After cooling, the Teflon bomb was evaporated to incipient dryness at 140 ℃. Then, 1 ml HNO_3 was added and the mixture was evaporated to dryness again. Subsequently, 1 ml of HNO_3, 1 ml of MQ water and 1 ml of 1 ppm In were added. The Teflon bomb was then resealed and heated at 190 ℃ for over 12 hours. The final solution was transferred to a polyethylene bottle and diluted to 100 g by adding 2% HNO_3. The standard materials GSR-3, RGM-2, BHVO-2, and JA-2 were used for quality control and their analysis results were consistent with the reference values.

Zircon O isotope analysis

The analysis of zircon oxygen isotopes was conducted using the second SHRIMP II equipment (multicollector) at the Beijing SHRIMP Center, CAGS. The procedures and conditions for the analysis were similar to those outlined by Ickert et al. [19] and Wan et al. [20]. The Cs^+ primary ion beam's intensity was approximately 3 nA, which resulted in secondary $^{16}O^{1-}$ count rates exceeding 10^9 cps. The diameters of the spots analyzed were 20 μm. The reference material used for calibrating instrumental mass fractionation (IMF) was the TEMORA 2 zircon. This reference material was mounted alongside the unknown samples. The standard was analyzed either two or three times at the beginning of each analytical session, and then after every third analysis of the unknown samples. The analyses results of zircon standards TEMORA (weighted $\delta^{18}O$ = 8.22 ± 0.11 ‰) are consistent with the recommended value ($\delta^{18}O$ = 8.20‰)[9]. The uncertainties associated with individual analyses are reported at the 1 sigma level.

Supplementary Notes

Sample descriptions and zircon U–Pb–Hf–O isotopic results

The newly discovered Baishan nucleus was primary composed of the 3.3–3.2 Ga monzogranite, *c.* 2.72 Ga monzogranite, *c.* 2.63 Ga monzogranite, and *c.* 2.5 Ga potassic granites (monzogranite and syenogranite). Encircling the Baishan nucleus are *c.* 2.5 Ga potassic granites, TTG, and supracrustal sequences (Supplementary Fig. 1). To establish the spatial-temporal framework of the Baishan nucleus, a total of 21 geochronological

samples were collected, with their locations depicted in Supplementary Fig. 1. Detailed descriptions of outcrops, mineral assemblages, along with interpretations of zircon U–Pb and Hf–O isotopes are as follows.

c. 3.3 Ga monzogranite (22BS20-3) with 3.6–3.5 Ga xenocrystic zircons

Sample 22BS20-3 was collected as a xenolith within the *c.* 3.23 Ga monzogranite, with a diffuse contact occurring between both lithologies (Supplementary Fig. 2a, b). The *c.* 3.3 Ga monzogranite exhibits weak gneissic schistosity. The mineral assemblage is composed of plagioclase, quartz, hornblende, and minor biotite, along with other accessory minerals (Supplementary Fig. 2c). Most of the grains are either subhedral or anhedral, with sizes ranging from 1 to 3 mm. Both SHRIMP and LA–ICP–MS methods were used to analyze Sample 22BS20-3, and both analyses yielded similar zircon U–Pb ages. The results of the LA–ICP–MS zircon U–Pb dating are listed in Supplementary Data 1, while the results from the SHRIMP analysis can be found in Supplementary Data 2. The zircons of Sample 22BS20-3 exhibit core-rim or core-mantle-rim structures in CL images (Supplementary Fig. 5a, b). The cores display concentric oscillatory zones with high Th/U ratios (0.11–0.81, except three analyses of 0.01, 0.07, and 0.08) suggestive of a magmatic origin. Conversely, zircon rims and mantles are relatively luminescent or lack interior structures, indicating a metamorphic origin. Analyses on zircon cores can be divided into two groups. The older age group dated by LA–ICP–MS and SHRIMP yield $^{207}Pb/^{206}Pb$ ages of 3570–3364 Ma and 3573–3283 Ma with upper intercept ages of 3558 ± 61 Ma and 3546 ± 20 Ma, respectively. The four concordant analyses conducted by SHRIMP yielded a weighted mean age of 3571 ± 2 Ma (MSWD = 1.06), which is interpreted as the age of xenocrystic zircons. The younger age group dated by LA–ICP–MS and SHRIMP yield $^{207}Pb/^{206}Pb$ ages of 3239–3076 Ma and 3252–2917 Ma with upper intercept ages of 3271 ± 25 Ma and 3238 ± 45 Ma, respectively. This younger c. 3.3 Ga is interpreted as the crystallization age for Sample 22BS20-3. Additionally, several analyses from either mantles or rims yield $^{207}Pb/^{206}Pb$ ages of 2756–2559 Ma, as revealed by SHRIMP dating. Other analyses from the rims conducted by LA–ICP–MS and SHRIMP yield $^{207}Pb/^{206}Pb$ ages of 2547–2340 Ma and 2527–2460 Ma respectively. These analyses yield a weighted mean age of 2508 ± 9 Ma (MSWD = 2.7; n = 9), which is interpreted as the latest metamorphic age for Sample 22BS20-3.

A total of 25 *in situ* Lu–Hf and 31 *in situ* oxygen isotopic analyses were performed at the same location as the SHRIMP dating. The 3.6–3.5 Ga zircons have ε_{Hf} value ranging from −3.5 to 0.0, with a weight mean value of −1.4 ± 0.7 (2SE; n = 12) (Supplementary Data 3). These zircons possess T_{DM}^2 ages between 4146 and 3931 Ma, and their $\delta^{18}O$ values vary from 5.08 to 6.27‰, with a weighted mean value of 5.8 ± 0.2‰ (2SE; n = 17) (Supplementary Data 2). In contrast, the *c.* 3.3 Ga zircons display more evolved ε_{Hf} values (−5.2 to −2.0) and slightly elevated $\delta^{18}O$ values of 5.23–7.69‰, with a weighted mean value of 6.6 ± 0.4‰ (Supplementary Data 2, 3). Notably, these *c.* 3.3 Ga zircons have similar ancient T_{DM}^2 ages (4115–3851 Ma) to the *c.* 3.6 Ga zircons. Meanwhile, thirteen *in situ* Lu–Hf analyses were conducted at the same locations as LA–ICP–MS dating work. The zircons, with $^{207}Pb/^{206}Pb$ ages of 3527–3076 Ma, exhibit ε_{Hf} value ranging from −6.6 to −1.6. These zircons have T_{DM}^2 ages from 4255 to 3951 Ma (Supplementary Data 3), which align with the results from the SHRIMP dating grains analysis.

c. 3.23 Ga monzogranite with 3.6–3.5 Ga xenocrystic zircons

Two samples used for LA–ICP–MS dating (21LJ38-1 and Z2010-3) were collected from the *c.* 3.23 Ga monzogranite, which serves as the host rock of the *c.* 3.3 Ga monzogranite (Supplementary Fig. 2a). Both samples exhibit similar mineral assemblages, primarily consisting of quartz, plagioclase, microcline, and biotite, along with minor hornblende and other accessory minerals (Supplementary Fig. 2d–g). Most zircons in these samples display core-rim structures (Supplementary Fig. 5c, d), with the cores exhibiting clear concentric oscillatory zones indicative of a magmatic origin. The LA–ICP–MS U–Pb dating results for these zircon cores can be divided into two age groups: an older group that has undergone significant Pb loss with variable $^{207}Pb/^{206}Pb$ ages between 3572 and 3328 Ma (Supplementary Data 1), considered to be inherited from their source rocks; and a relatively younger group with $^{207}Pb/^{206}Pb$ ages ranging from 3255 to 3186 Ma. This younger group has an upper intercept age of

3230 ± 3 Ma (MSWD = 1.4; n = 13) and weighted mean ages of 3220 ± 15 Ma (MSWD = 0.71; n = 9) for samples 21LJ38-1 and Z2010-3, respectively (Supplementary Fig. 5c, d). Analyses on zircon rims or re-crystallized zircons yield a range of $^{207}Pb/^{206}Pb$ ages from 2555 to 2468 Ma (Supplementary Data 1), with upper intercept ages of 2518 ± 9 Ma (MSWD = 2.7; n = 15) and 2512 ± 9 Ma (MSWD = 0.92; n = 13) for samples 21LJ38-1 and Z2010-3, respectively (Supplementary Fig. 5c, d). Of which, thirteen analyses from Sample Z2010-3 yield a weighted mean age of 2505 ± 13 Ma (MSWD = 0.52; n = 13). These c. 2.5 Ga ages are interpreted to date the timing of metamorphism of the *c.* 3.23 Ga monzogranite. Furthermore, several analyses yield concordant $^{207}Pb/^{206}Pb$ ages ranging from 3112 to 2634 Ma, which may represent mixing ages (Supplementary Fig. 5c, d). A total of fifteen 3.6–3.5 Ga xenocrystic zircons were analyzed for Lu–Hf isotopes. They have ε_{Hf} values ranging from −3.6 to 0.1, with mean values of −1.9 and −0.3 for Sample 21LJ38-1 and Z2010-3 respectively (Supplementary Data 6). Nineteen *c.* 3.23 Ga zircons display concentrated Hf isotopes, with ε_{Hf} values of −6.4 to −2.8 (except for two analyses of −1.5 and −2.1). The *c.* 3.23 Ga zircons and the ancient xenocrystic zircons have similar T_{DM}^2 ages ranging from 4243 to 3949 Ma (except for two analyses of 3827 and 3867).

c. 2.72 Ga monzogranite (22BS15-1)

Sample 22BS15-1 was collected from the *c.* 2.72 Ga monzogranite, which is difficult to distinguish from other monzogranites of varying ages in the field. The *c.* 2.72 Ga monzogranite has a massive structure with a mineral assemblage of quartz, plagioclase, microcline, as well as minor biotite (Supplementary Fig. 2h-j). These grains predominantly range in size from 1 to 5 mm. The zircons of Sample 22BS15-1 exhibit typical core-rim structures; the cores display concentric oscillatory zoning while the rims are luminescent and lack interior structures (Supplementary Fig. 5e, f). Both LA–ICP–MS and SHRIMP methods were employed to constrain the crystallization ages of Sample 22BS15-1 (Supplementary Data 1, 2). SHRIMP U-Pb dating suggests that the cores yield similar $^{207}Pb/^{206}Pb$ ages with a weighted mean age of 2713 ± 6 Ma (MSWD = 1.8; n = 11), while analyses of the rims yield a weighted mean age of 2497 ± 13 Ma (MSWD = 2.7; n = 6). LA–ICP–MS U-Pb dating results suggest a similar weighted mean $^{207}Pb/^{206}Pb$ age (2722 ± 6 Ma; MSWD = 0.37; n = 19) for zircon cores (Supplementary Fig. 5e). Consequently, the *c.* 2.72 Ga and *c.* 2.5 Ga ages are interpreted as crystallization and metamorphic ages, respectively. A total of 32 zircon grains were analyzed for Lu–Hf isotopes (Supplementary Data 3), specifically, 12 zircon grains from SHRIMP target and 20 grains from LA–ICP–MS target. These zircons exhibit negative ε_{Hf} values ranging from −7.9 to −5.0, except for two analyses that yielded values of −4.5 and −4.2. They also possess ancient T_{DM}^2 ages from 4032 to 3823 Ma, excluding two analyses that resulted in ages of 3743 and 3714 Ma. Oxygen isotope analyses conducted on eleven zircons indicate $\delta^{18}O$ values ranging from 4.56 to 6.60‰ (Supplementary Data 2), with a weighted mean value of 5.9 ± 0.3‰, a characteristic similar to zircons of mantle origin.

c. 2.63 Ga monzogranite (22BS19-1)

Sample 22BS19-1 is a monzogranite with massive structure. It primarily consists of quartz, plagioclase, and microcline, with minor amounts of biotite (Supplementary Fig. 2k, l). The contact relationship between the *ca* 2.63 Ga monzogranite and other geological units remains unknown due to extensive vegetation and sedimentary cover. A total of 40 analyses were conducted using the LA–ICP–MS method. Nineteen analyses yield an upper intercept age of 2627 ± 12 Ma (MSWD = 1.08; n = 19). Among these, 11 analyses with minor discordance yield a weighted mean age of 2627 ± 16 Ma (MSWD = 0.43; n = 11) (Supplementary Fig. 5g). Therefore, it can be inferred that the sample crystallized around approximately 2627 Ma. Sixteen zircon Lu-Hf isotopic analyses indicate that they possess unradiogenic Hf isotopes, with negative ε_{Hf} values ranging from −4.4 to −2.0, excluding two analyses that yielded values of −0.5 and −1.0. The T_{DM}^2 ages for these zircons span from 3681 to 3478 Ma, except for two analyses that resulted in ages of 3334 and 3381 Ma (Supplementary Data 3).

c. 2.5 Ga potassic granites

The *c.* 2.5 Ga potassic granites constitute the majority of the Baishan nucleus and are primarily composed of monzogranite and syenogranite. Sixteen geochronological samples were collected for LA–ICP–MS dating; these include monzogranite samples such as 21LJ39-1 and syenogranite samples like 22BS27-1 (Supplementary Figs 3, 4). These *c.* 2.5 Ga potassic granite samples exhibit similar mineral assemblages comprising quartz, plagioclase, microcline and minor biotite—with or without hornblende—but the monzogranites contain more plagioclase and fewer microclines compared to the syenogranites (Supplementary Figs 3, 4). Among these samples, 21LJ39-1, 21LJ35-1, and 22BS16-1 contain an abundance of ancient xenocrystic zircons with $^{207}Pb/^{206}Pb$ ages ranging from 3665 to 2611 Ma (Supplementary Data 1, 2, and Supplementary Fig. 5h-k). Notably, two xenocrystic zircons exhibit concordant $^{207}Pb/^{206}Pb$ ages of 3663 ± 20 Ma (1σ) and 3665 ± 8 Ma (1σ) (Supplementary Fig. 5j), potentially indicating the presence of Eoarchean continental crust in this region. The youngest age groups from these samples yield similar $^{207}Pb/^{206}Pb$ ages approximately between 2530 to 2500 Ma (Supplementary Fig. 5h-k), representing the timing of crystallization. The remaining geochronologic samples, which contain rare xenocrystic zircons, exhibit $^{207}Pb/^{206}Pb$ weighted mean ages or upper intercept ages ranging from 2551 to 2454 Ma (Supplementary Fig. 6).

The xenocrystic zircons, with ages ranging from 3665 to 3462 Ma, possess sub-chondritic Hf isotopes characterized by negative ε_{Hf} values from −2.9 to −0.4 (Supplementary Data 3). In contrast, younger xenocrystic zircons, with ages between 3317 and 2654 Ma, exhibit more evolved Hf isotopes with negative ε_{Hf} values ranging from −10.2 to −3.1. The T_{DM}^2 ages for these xenocrystic zircons span from 4285 to 3887 Ma, barring one analysis that extends up to 4489 Ma (Supplementary Data 3). This range is broadly consistent with that of the 3.6–3.5 Ga zircons within the 3.3–3.2 Ga monzogranites. The c. 2.5 Ga zircons can be categorized into two distinct groups based on their Hf isotopes. One group possesses radiogenic Hf isotopes, characterized by positive ε_{Hf} values, while the other group exhibits unradiogenic Hf isotopes, denoted by negative ε_{Hf} values. The first group includes samples from the outer region of the Baishan nucleus (22BS29-1, 22BS23-1, 21LJ18-1, 21LJ19-1, 22BS18-4, 22BS28-1, 23HX22-1, and 21LJ06-3). These samples have weighted mean ε_{Hf} values ranging from 1.0 to 4.7 and corresponding T_{DM}^2 ages between 3148 to 2834 (Supplementary Data 6). In contrast, the second group comprises samples primarily from the core of the BLC (21LJ39-1, 21LJ35-1, 21LJ16-1, 22BS32-1, 23HX23-1, 23HX28-1, and 23HX29-1). These samples display weighted mean ε_{Hf} values from −3.6 to −1.1 and T_{DM}^2 ages spanning 3535 to 3224 (Supplementary Data 6). Additionally, oxygen isotopes were analyzed in the zircons of Sample 21LJ39-1 (Supplementary Data 2). Three xenocrystic zircons aged 3567–3488 Ma exhibit $\delta^{18}O$ values between 5.50–6.48‰, with a weighted mean value of 6.1 ± 0.6 ‰ (2SE). The Mesoarchean xenocrystic zircons (3189–2949 Ma) display $\delta^{18}O$ values ranging from 5.79–7.17‰, with a weighted mean value of 6.5 ± 0.3 ‰ (2SE), barring one analysis at 3.91‰. Lastly, the *c.* 2.5 Ga zircons from Sample 21LJ39-1 have $\delta^{18}O$ values between 4.91 to 6.57‰, with a weighted mean value of 5.7 ± 0.2 ‰ (2SE), excluding three abnormal analyses at 2.08‰, 3.08‰, and 4.33‰.

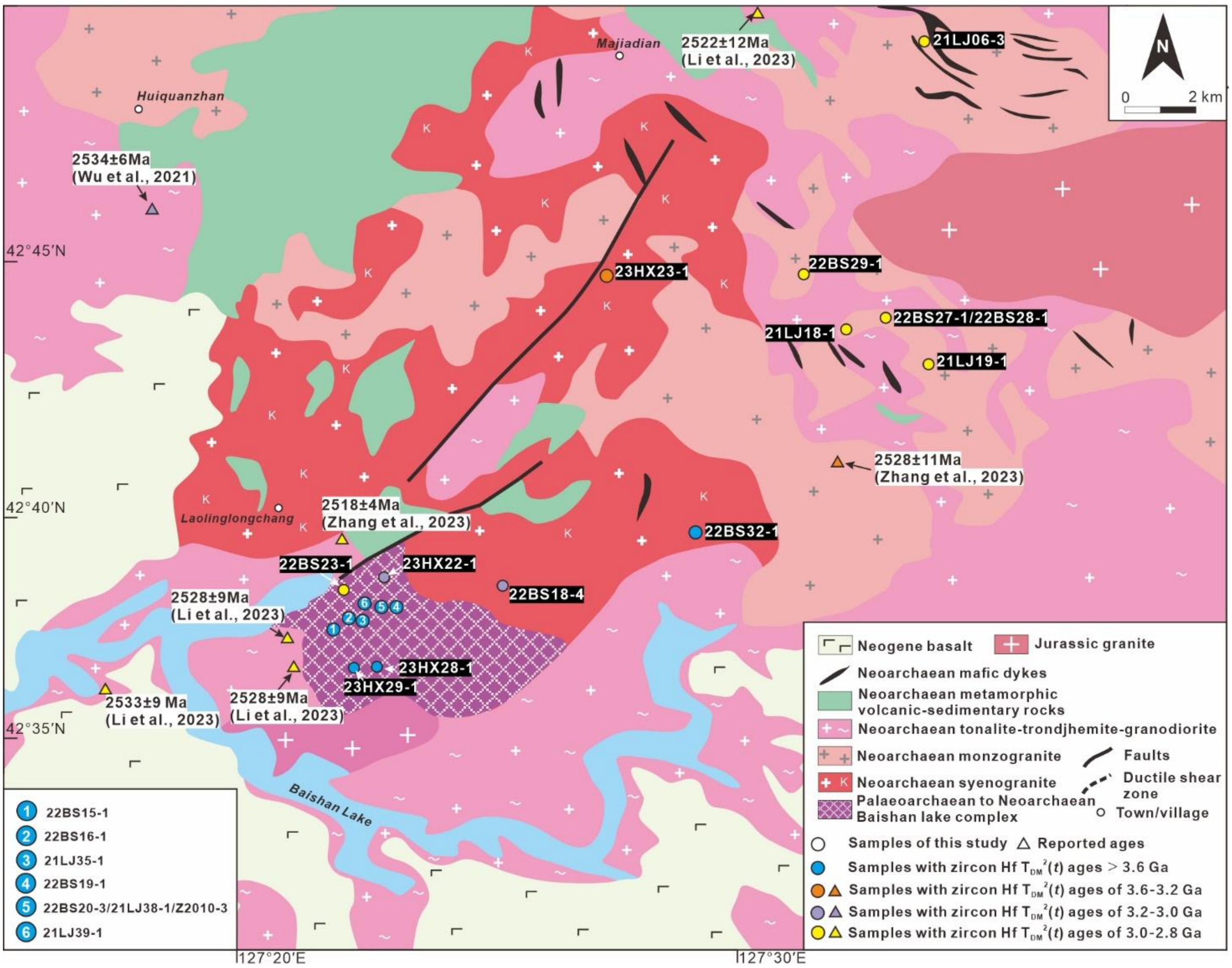

Supplementary Fig. 1 Geological map of the Baishan Lake complex and its surrounding area, showing the locations of geochronological samples. Blue circles with corresponding numbers represent the locations of specific samples, which including: ① Sample 22BS15-1; ② Sample 22BS16-1; ③ Sample 21LJ35-1; ④ Sample 22BS19-1; ⑤ Samples 22BS20-3, 21LJ38-1, Z2010-3; ⑥ Sample 21LJ39-1. Cited age data are from Wu et al. [21], Li et al. [22].

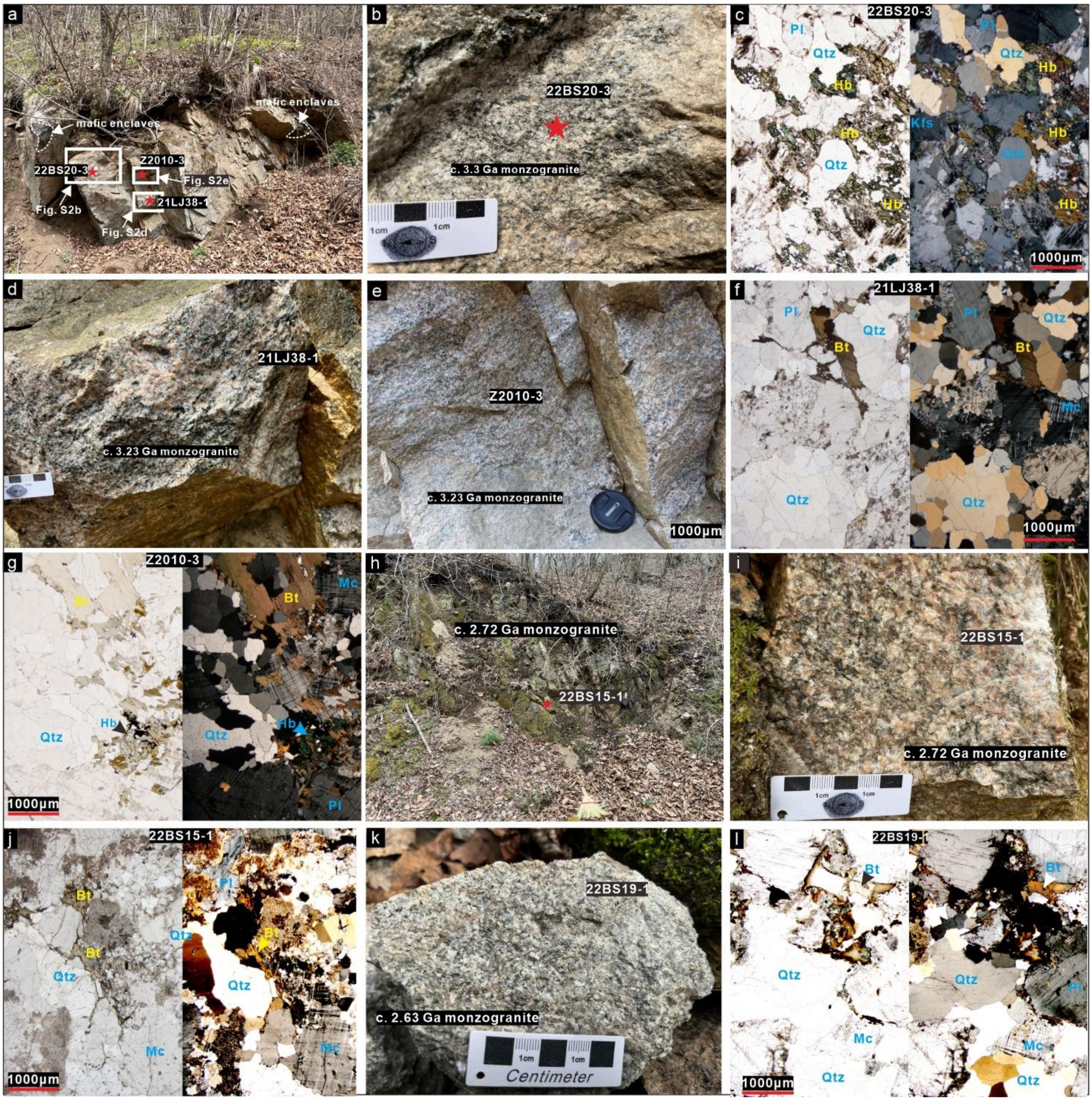

Supplementary Fig. 2 Field, macro-microscopic images of Archean granitoids from the Baishan Lake complex. (a) Xenolithic occurrence of *c.* 3.3 Ga monzogranite within the surrounding *c.* 3.23 Ga monzogranites. (b, c) Macroscopic petrography and photomicrograph of the *c.* 3.3 Ga monzogranite. (d-g) Macroscopic petrography and photomicrograph of the *c.* 3.23 Ga monzogranites. (h-j) Outcrop, macroscopic petrography, and photomicrograph of the *c.* 2.72 Ga monzogranite. (k, l) Macroscopic petrography and photomicrograph of the *c.* 2.63 Ga monzogranite. Mineral abbreviations: Bt - biotite, Hb - Hornblende, Kfs - K-feldspar, Mc - Microcline, Pl - Plagioclase, Qtz - Quartz.

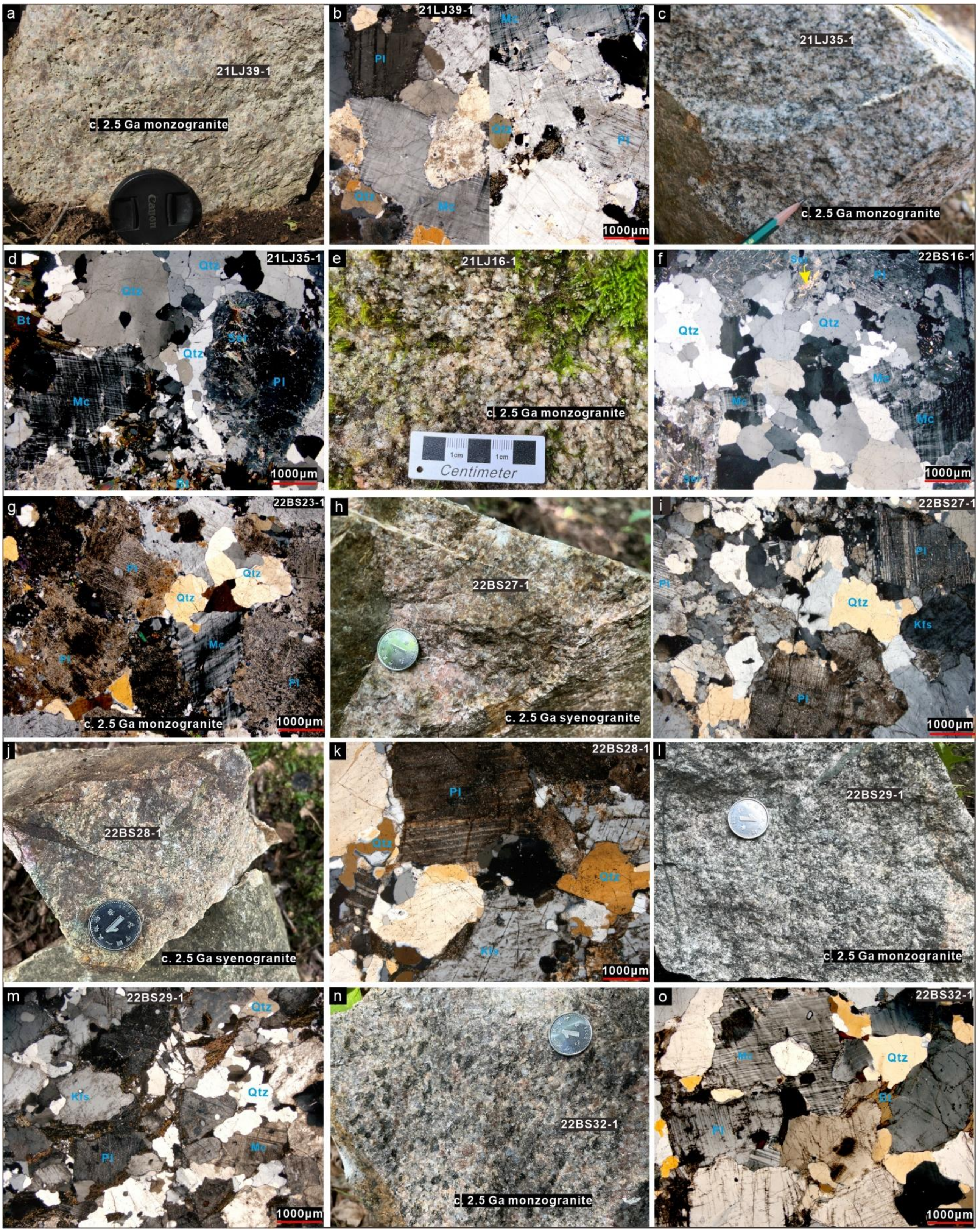

Supplementary Fig. 3 Macro-microscopic images of Neoarchean potassic granites from the Baishan Lake complex. (a, b) Macroscopic petrography and photomicrograph of Sample 21LJ39-1. (c, d) Macroscopic petrography and photomicrograph of Sample 21LJ35-1. (e, f) Macroscopic petrography and photomicrograph of Sample 21LJ16-1. (g) Photomicrograph of Sample 22BS32-1. (h, i) Macroscopic petrography and photomicrograph of Sample 22BS27-1. (j, k) Macroscopic petrography and photomicrograph of Sample 22BS28-1. (l, m) Macroscopic petrography and photomicrograph of Sample 22BS29-1. (n, o) Macroscopic petrography and photomicrograph of Sample 22BS32-1. Mineral abbreviations: Bt - biotite, Hb - hornblende, Kfs - K-feldspar, Mc - microcline, Pl - plagioclase, Qtz - quartz, Ser - sericite.

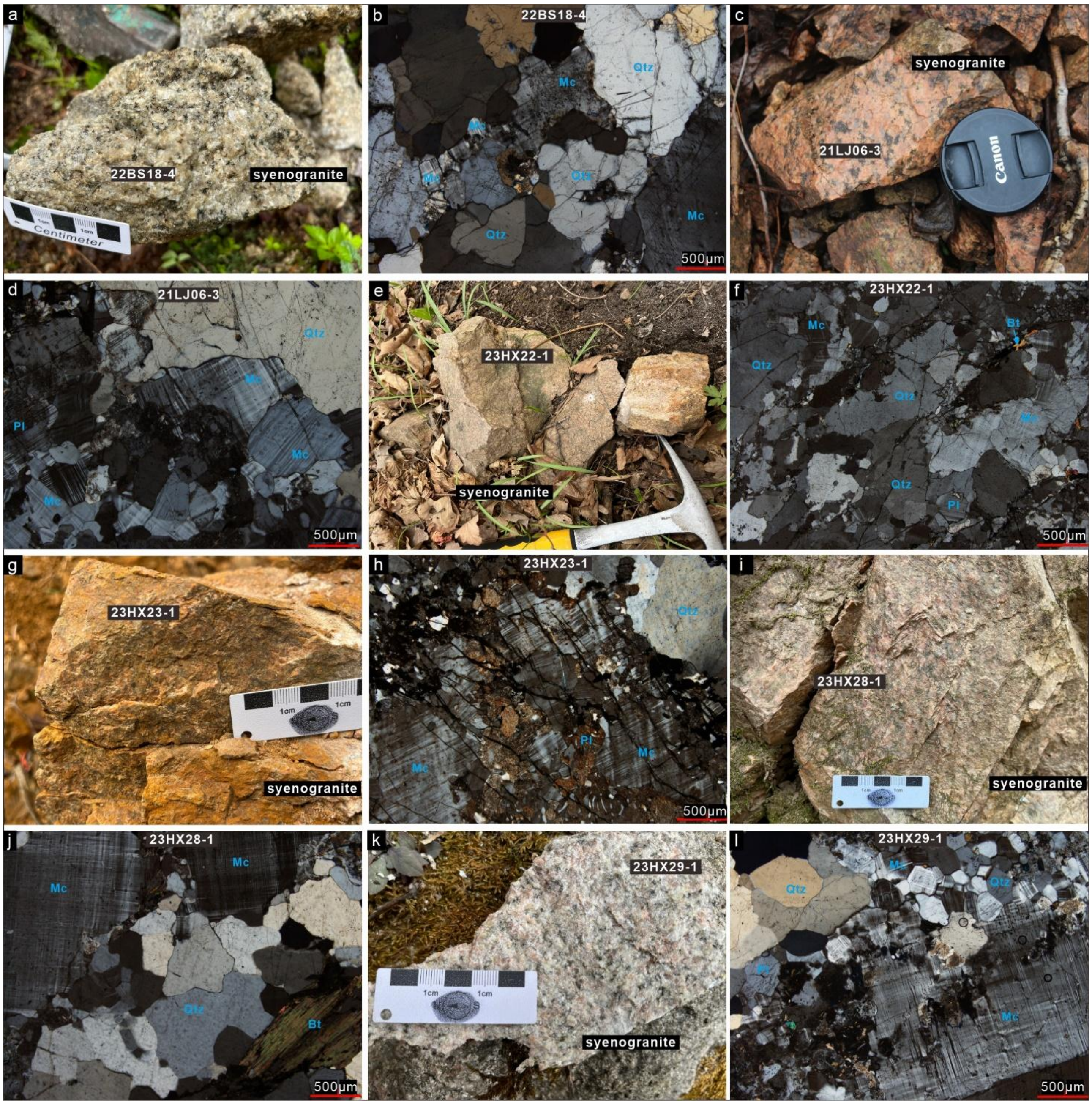

Supplementary Fig. 4 Macro-microscopic images of Neoarchean syenogranites from the Baishan Lake complex. (a, b) Macroscopic petrography and photomicrograph of Sample 22BS18-4. (c, d) Macroscopic petrography and photomicrograph of Sample 21LJ06-3. (e, f) Macroscopic petrography and photomicrograph of Sample 23HX22-1. (g, h) Photomicrograph of Sample 23HX23-1. (i, j) Macroscopic petrography and photomicrograph of Sample 23HX28-1. (k, l) Macroscopic petrography and photomicrograph of Sample 23HX29-1. Mineral abbreviations: Bt - biotite, Mc - microcline, Pl - plagioclase, Qtz - quartz.

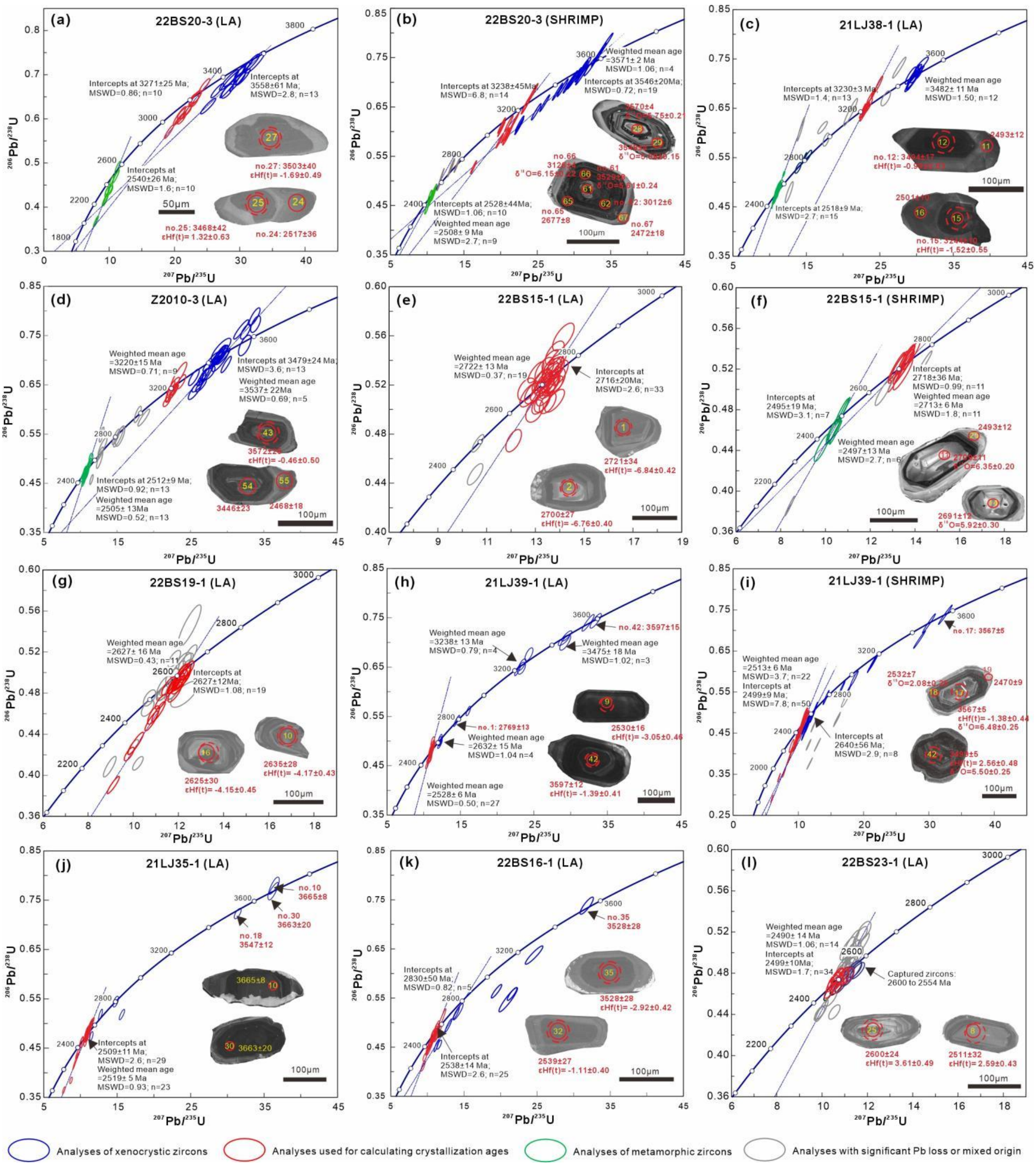

Supplementary Fig. 5 Zircon concordia diagram and representative zircon cathodoluminescence (CL) images of the Archean granitoids in the BLC. The positions of U-Pb and O analyses, indicated by red solid ellipses, along with Hf analyses, denoted by red dashed ellipses, are illustrated in the CL images. Red and green ellipses in the concordia diagram represent analyses that were used for calculating crystallization and metamorphism ages, respectively. Blue ellipses represent analyses of xenocrystic zircons. Grey ellipses in the concordia diagram represent analyses that were excluded from age calculation due to significant Pb loss or mixed origin. All these representative zircons exhibit core-rim structures in CL images. The cores show concentric oscillatory zones, while zircon rims are relatively luminescent or lack interior structures.

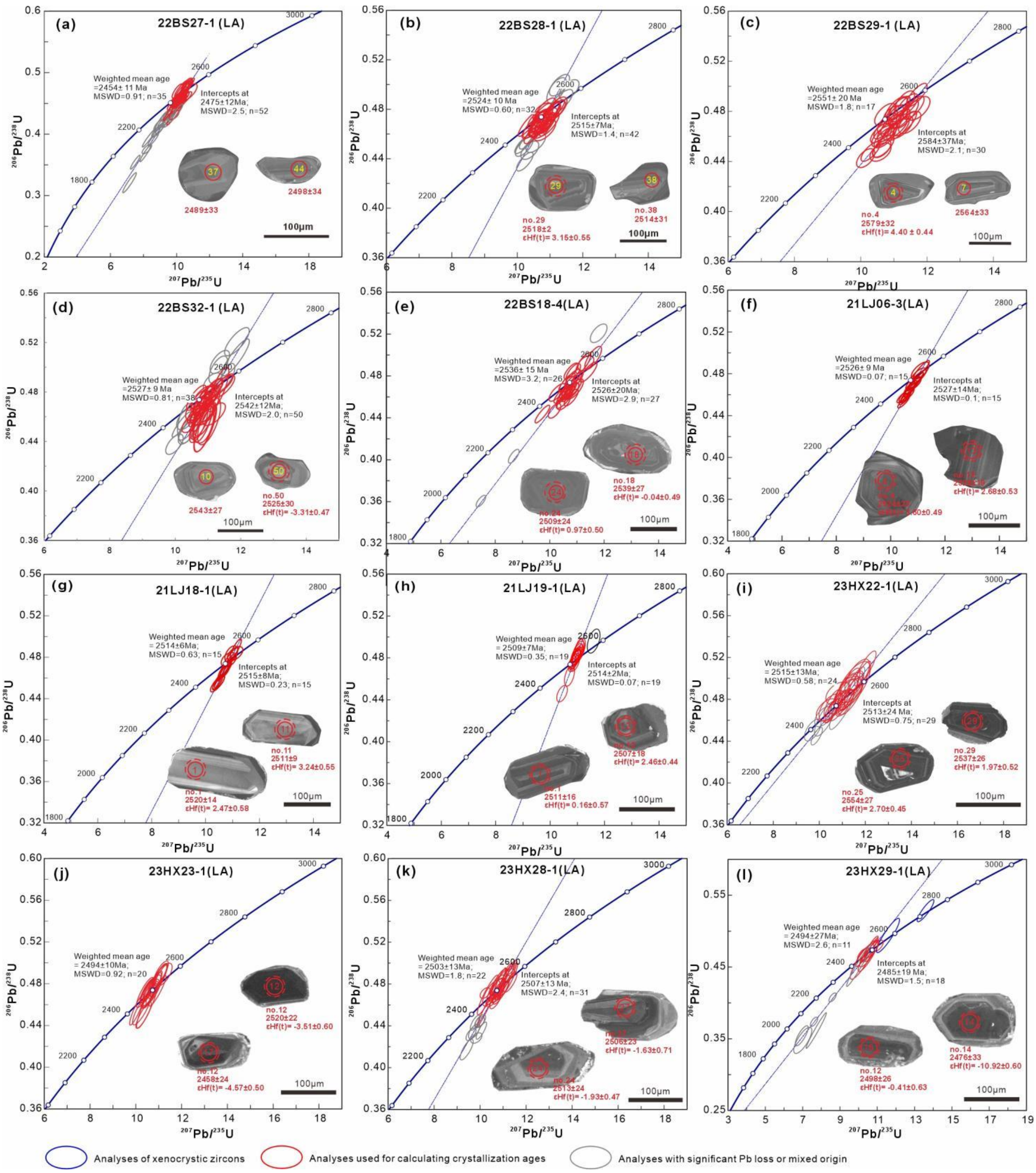

Supplementary Fig. 6 Zircon concordia diagram and representative zircon cathodoluminescence (CL) images of the Archean granitoids in the BLC. The positions of U-Pb and O analyses, indicated by red solid ellipses, along with Hf analyses, denoted by red dashed ellipses, are illustrated in the CL images. Red ellipses in the concordia diagram represent analyses that were used for calculating crystallization ages. Blue ellipses represent analyses of xenocrystic zircons. Grey ellipses in the concordia diagram represent analyses that were excluded from age calculation due to significant Pb loss or mixed origin. All these representative zircons exhibit concentric oscillatory zones, and some of them have narrow rims.

Reference

1. Liu, Y. et al. Continental and Oceanic Crust Recycling-induced Melt-Peridotite Interactions in the Trans-North China Orogen: U-Pb Dating, Hf Isotopes and Trace Elements in Zircons from Mantle Xenoliths. *Journal of Petrology* **51**, 537-571 (2010).
2. Wiedenbeck, M. et al. Three Natural Zircon Standards for U-Th-Pb, Lu-Hf, Trace Element and Ree Analyses. *Geostandards and Geoanalytical Research* **19**, 1-23 (1995).
3. Sláma, J. et al. Plešovice zircon — A new natural reference material for U–Pb and Hf isotopic microanalysis. *Chemical Geology* **249**, 1-35 (2008).
4. Yuan, H. et al. Accurate U-Pb Age and Trace Element Determinations of Zircon by Laser Ablation-Inductively Coupled Plasma-Mass Spectrometry. *Geostandards and Geoanalytical Research* **28**, 353-370 (2004).
5. Thompson, J.M., Meffre, S. & Danyushevsky, L. Impact of air, laser pulse width and fluence on U–Pb dating of zircons by LA-ICPMS. *Journal of Analytical Atomic Spectrometry* **33**, 221-230 (2018).
6. Paton, C. et al. Improved laser ablation U-Pb zircon geochronology through robust downhole fractionation correction. *Geochemistry, Geophysics, Geosystems* **11**, n/a-n/a (2010).
7. Ludwig, K.R. Isoplot v. 4.15: a geochronological toolkit for Microsoft Excel. *Berkeley Geochron. Center Sp. Publ* **4**, 75 (2011).
8. Williams, I.S. in Applications of Microanalytical Techniques to Understanding Mineralizing Processes, Vol. 7 1-35 (Society of Economic Geologists, 1997).
9. Black, L.P. et al. Improved 206Pb/238U microprobe geochronology by the monitoring of a trace-element-related matrix effect; SHRIMP, ID–TIMS, ELA–ICP–MS and oxygen isotope documentation for a series of zircon standards. *Chemical Geology* **205**, 115-140 (2004).
10. Ludwig, K.R. User's Manual for SQUID 2. *Berkeley Geochronology Center Special Publication, Berkeley* **5**, 1-110 (2009).
11. Hu, Z. et al. Improved in situ Hf isotope ratio analysis of zircon using newly designed X skimmer cone and jet sample cone in combination with the addition of nitrogen by laser ablation multiple collector ICP-MS. *Journal of Analytical Atomic Spectrometry* **27**, 1391-1399 (2012).
12. Woodhead, J., Hergt, J., Shelley, M., Eggins, S. & Kemp, R. Zircon Hf-isotope analysis with an excimer laser, depth profiling, ablation of complex geometries, and concomitant age estimation. *Chemical Geology* **209**, 121-135 (2004).
13. Fisher, C.M., Vervoort, J.D. & Hanchar, J.M. Guidelines for reporting zircon Hf isotopic data by LA-MC-ICPMS and potential pitfalls in the interpretation of these data. *Chemical Geology* **363**, 125-133 (2014).
14. Blichert-Toft, J., Chauvel, C. & Albarède, F. Separation of Hf and Lu for high-precision isotope analysis of rock samples by magnetic sector-multiple collector ICP-MS. *Contributions to Mineralogy and Petrology* **127**, 248-260 (1997).
15. Zhang, W. & Hu, Z. Estimation of Isotopic Reference Values for Pure Materials and Geological Reference Materials. *Atomic Spectroscopy* **41**, 93-102 (2020).
16. Scherer, E., Münker, C. & Mezger, K. Calibration of the Lutetium-Hafnium Clock. *Science* **293**, 683-687 (2001).
17. Blichert-Toft, J. & Albarède, F. The Lu-Hf isotope geochemistry of chondrites and the evolution of the mantle-crust system. *Earth and Planetary Science Letters* **148**, 243-258 (1997).
18. Griffin, W.L. et al. Zircon chemistry and magma mixing, SE China: In-situ analysis of Hf isotopes, Tonglu and Pingtan igneous complexes. *Lithos* **61**, 237-269 (2002).
19. Ickert, R.B. et al. Determining high precision, in situ, oxygen isotope ratios with a SHRIMP II: Analyses of MPI-DING silicate-glass reference materials and zircon from contrasting granites. *Chemical Geology* **257**, 114-128 (2008).
20. Wan, Y. et al. Extreme zircon O isotopic compositions from 3.8 to 2.5 Ga magmatic rocks from the Anshan area, North China Craton. *Chemical Geology* **352**, 108-124 (2013).
21. Wu, M., Lin, S., Wan, Y., Gao, J.-F. & Stern, R.A. Episodic Archean crustal accretion in the North China Craton:

Insights from integrated zircon U-Pb-Hf-O isotopes of the Southern Jilin Complex, northeast China. *Precambrian Research* **358**, 106150 (2021).

22. Li, Z. et al. Late Neoarchean TTG and monzogranite in the northeastern North China Craton: Implications for partial melting of a thickened lower crust. *Gondwana Research* **115**, 201-223 (2023).
