# Supplementary material for: Archaean multi-stage magmatic underplating drove formation of continental nuclei in the North China Craton
Source: Nat Commun. 2024 Jul 24;15:6231. doi: 10.1038/s41467-024-50435-5 (PMC11266541; doi:10.1038/s41467-024-50435-5)
Supplement: Supplementary file 3 — Description of Additional Supplementary Files [file 41467_2024_50435_MOESM3_ESM.pdf]

<u>Description of Additional Supplementary Files</u>

Supplementary Data 1. LA-ICP-MS zircon U-Pb ages of Palaeo-Neoarchaean granitoids from the Baishan nucleus, North China Craton. Data collection procedures are outlined in Supplementary Information.

Supplementary Data 2. Zircon SHRIMP U-Pb ages and O isotopes of Palaeo-Neoarchaean granitoids from the Baishan nucleus, North China Craton. Data collection procedures are outlined in Supplementary Information.

Supplementary Data 3. Zircon Lu-Hf isotopic data of Palaeo-Neoarchaean granitoids from the Baishan nucleus and standard materials. Data collection procedures are outlined in Supplementary Information.

Supplementary Data 4. Compilation of individual Archaean zircon Hf isotopes from the North China Craton and other cratons. Samples in grey are heterogeneous with p values ＜0.05. Sources of data are listed below the table.

Supplementary Data 5. Compilation of Archaean zircon O isotopes from the North China Craton and other cratons. Sources of data are listed below the table.

Supplementary Data 6. Mean values of Archaean zircon Hf isotopes from the North China Craton. Samples in grey are heterogeneous with p values ＜0.05. Sources of data are listed below the table.

Supplementary Data 7. Major and trace element concentrations of 3.2-2.5 Ga potassic granites from the Baishan nucleus, North China Craton. Data collection procedures are outlined in Supplementary Information. Major elements are reported in wt.%, trace and REE concentrations are reported in ppm.
